# An autophagy-related long non-coding RNA prognostic signature accurately predicts survival outcomes in bladder urothelial carcinoma patients

**DOI:** 10.18632/aging.103718

**Published:** 2020-08-15

**Authors:** Zhuolun Sun, Changying Jing, Chutian Xiao, Tengcheng Li

**Affiliations:** 1 Department of Urology, The Third Affiliated Hospital, Sun Yat-sen University, Guangzhou, 510630, China; 2 The Second Clinical Medical College, Shandong University of Traditional Chinese Medicine, Jinan, Shandong, China; 3 Equal contribution

**Keywords:** autophagy, bladder urothelial carcinoma, long non-coding RNA, prognostic signature, the cancer genome atlas

## Abstract

In this study, we analyzed the prediction accuracy of an autophagy-related long non-coding RNA (lncRNA) prognostic signature using bladder urothelial carcinoma (BLCA) patient data from The Cancer Genome Atlas (TCGA) database. Univariate and multivariate Cox regression analyses showed significant correlations between five autophagy-related lncRNAs, LINC02178, AC108449.2, Z83843.1, FAM13A-AS1 and USP30−AS1, and overall survival (OS) among BCLA patients. The risk scores based on the autophagy-related lncRNA prognostic signature accurately distinguished high- and low-risk BCLA patients that were stratified according to age; gender; grade; and AJCC, T, and N stages. The autophagy-related lncRNA signature was an independent prognostic predictor with an AUC value of 0.710. The clinical nomogram with the autophagy-related lncRNA prognostic signature showed a high concordance index of 0.73 and accurately predicted 1-, 3-, and 5-year survival times among BCLA patients in the high- and low-risk groups. The lncRNA-mRNA co-expression network contained 77 lncRNA-mRNA links among 5 lncRNAs and 49 related mRNAs. Gene set enrichment analysis showed that cancer- and autophagy-related pathways were significantly enriched in the high-risk group, and immunoregulatory pathways were enriched in the low-risk group. These findings demonstrate that an autophagy-related lncRNA signature accurately predicts the prognosis of BCLA patients.

## INTRODUCTION

Bladder urothelial carcinoma (BLCA) is the most common malignant tumor of the urinary system, accounting for 6.6% and 2.1% of the total cancer patients among men and women in the world, respectively [1, 2]. Patients with transitional cell carcinoma account for approximately 90% of all BCLA cases [3]. Despite great strides in radiotherapy, surgery, and adjuvant chemotherapy, the survival outcomes remain poor for BCLA patients, with approximately 30% of the patients diagnosed with advanced muscle-invasive disease [2]. Moreover, the current clinical staging system requires improvement in accurately predicting the prognosis of BCLA patients [4].

Autophagy is an evolutionarily conserved catabolic process, which occurs at basal levels under normal conditions to eliminate worn out cellular organelles and damaged or mis-folded proteins [5]. However, dysregulation of autophagy is implicated in several human diseases, such as cancer [6], neurodegenerative disorders [7], cardiovascular diseases [8], and inflammatory disorders related to infectious diseases [9]. Autophagy is associated with tumor suppression or oncogenesis depending upon the stage of tumor development [10, 11]. Recent studies show that modulation of autophagy improves the sensitivity of BCLA tumors to chemotherapeutic agents [12, 13]. Hence, it is critical to discover autophagy-related biomarkers that can serve as valuable the early diagnostic and prognostic biomarkers for BCLA patients.

Genome sequencing studies show that nearly 90% of the human transcriptome represents non-coding RNA or ncRNA [14]. The long non-coding RNAs (lncRNAs) are a type of ncRNAs with transcripts of >200 nucleotides in length without any protein-coding capacity [15]. LncRNAs regulate important biological functions related to cell growth and survival, genomic imprinting, chromatin modifications, and allosteric regulation of enzyme activities [16]. Furthermore, pathogenesis of several human diseases including several different types of cancers involves dysregulation of specific lncRNAs [17]. Some studies have shown that lncRNAs regulate autophagic functions. For example, Ying et al. demonstrated that downregulation of lncRNA MEG3 promotes proliferation of bladder cancer cells by activating autophagy [18]. Another study shows that lncRNA MALAT1 regulates multi-drug resistance of hepatocellular carcinoma cells by altering autophagy [19]. LncRNA PVT1 promotes *in vitro* and *in vivo* pancreatic ductal adenocarcinoma progression by activating autophagy through its regulation of the miR-20a-5p/ULK1 axis [20].

New advances in genome sequencing technology and bioinformatics have helped to identify potential prognostic biomarkers that can predict survival outcomes in cancer patients [21, 22]. Therefore, we postulated that autophagy-related lncRNAs may be valuable prognostic biomarkers for BLCA patients. In this study, we systematically analyzed the relationship between the expression of autophagy-related lncRNAs and the clinicopathological characteristics of 409 BLCA patients from The Cancer Genome Atlas (TCGA) database. We also constructed a prognostic signature based on 5 autophagy-related lncRNAs and evaluated its ability to independently and accurately predict the prognosis of BLCA patients.

## RESULTS

### Identification of prognostically significant autophagy-related lncRNAs in BLCA patient tissue samples

**Figure 1:**
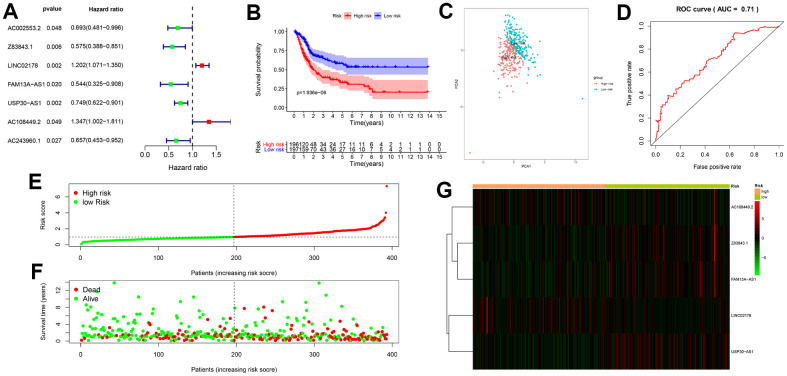
**Construction and validation of the autophagy-related lncRNA prognostic signature in BCLA patients.** (**A**) The univariate Cox regression analysis results show that 7 autophagy-related lncRNAs, AC002553.2, Z83843.1, LINC02178, FAM13A−AS1, USP30−AS1, AC108449.2 and AC243960.1, correlate with overall survival (OS) of BCLA patients from the TCGA database. (**B**) Kaplan–Meier survival curve analysis shows that survival time of patients with high-risk scores based on the autophagy-related lncRNA prognostic signature is significantly shorter than those with low-risk scores. (**C**) Principal components analysis (PCA) based on the confirmed five autophagy-related lncRNAs showed two significantly different distribution patterns between high-risk and low-risk groups. (**D**) Receiver operating characteristic (ROC) curve analysis shows the accuracy of the autophagy-related lncRNA prognostic signature in predicting survival times (prognosis) of BCLA patients from the TCGA database. (**E**) Distribution of risk scores of high- and low-risk BCLA patients based on the autophagy-related lncRNA prognostic signature. (**F**) Scatter plot shows the correlation between survival time and risk score of BCLA patients based on the autophagy-related lncRNA prognostic signature. (**G**) Heatmap shows that high-risk patients expressed higher levels of risk factors (AC108449.2 and LINC02178), while low-risk patients expressed higher levels of protective factors (Z83843.1, FAM13A−AS1 and USP30−AS1).

We identified 14153 lncRNAs by analyzing the RNA-seq data of the BLCA patient tissue samples from the TCGA database. We also extracted 232 autophagy-related genes from the Human autophagy database (HADb) analysis. We then identified 49 autophagy-related lncRNAs by performing Pearson correlation analysis between the lncRNAs and the autophagy-related genes using |R| > 0.7 and *P* < 0.05 as the selection criteria. Univariate Cox regression analysis of the 49 autophagy-related lncRNAs showed that expression of 7 lncRNAs, namely, AC002553.2, Z83843.1, LINC02178, FAM13A−AS1, USP30−AS1, AC108449.2 and AC243960.1 significantly correlated with the overall survival (OS) of BLCA patients (*P* < 0.05; Figure 1A). Multivariate Cox regression analysis showed that 5 of the 7 autophagy-related lncRNAs were good candidates for constructing the prognostic signature based on the lowest Akaike information criterion (AIC) (Table 1). Among the 5 autophagy-related lncRNAs that were included in the prognostic signature, LINC02178 and AC108449.2 were considered as risk factors with HR values greater than 1, whereas the remaining 3 lncRNAs, Z83843.1, FAM13A−AS1 and USP30−AS1, were considered as protective factors with HR values less than 1.

**Table 1:** Akaike information criterion for the models.

**Model**	**Prognostic signature combination**	**AIC**
1	AC002553.2 + Z83843.1 + LINC02178 + FAM13A-AS1 + USP30-AS1 + AC108449.2 + AC243960.1	1584.35
2	AC002553.2 + Z83843.1 + LINC02178 + FAM13A-AS1 + USP30-AS1 + AC108449.2	1582.81
3	Z83843.1 + LINC02178 + FAM13A-AS1 + USP30-AS1 + AC108449.2	1581.61

### Evaluation of the prognostic signature containing 5 autophagy-related lncRNAs

The risk score for each BCLA patient in the TCGA dataset was calculated using the following formula for the autophagy-related lncRNA signature: risk score = (-0.677 × expression level of Z83843.1) + (0.162 × expression level of LINC02178) + (-0.403 × expression level of FAM13A−AS1) + (-0.307 × expression level of USP30−AS1) + (0.489 × expression level of AC108449.2). Then, BLCA patients were divided into high-risk (n = 196) and low-risk (n = 197) groups using the median risk score (= 1.093) as the cut-off point. Kaplan-Meier survival curve analysis showed that the OS of BCLA patients with high-risk scores was significantly shorter than those with low-risk scores (Figure 1B). The 3-year survival rates were 39% and 64%, and the 5-year survival rates were 32% and 56% for the high-risk and low-risk patients, respectively. A principal components analysis (PCA) based on the five autophagy-related lncRNAs showed two significantly different distribution patterns between high-risk and low-risk groups (Figure 1C). Time-dependent receiver operating characteristic (ROC) curve analysis showed that the area under the ROC (AUC) value for the autophagy-related lncRNA prognosis signature was 0.710 (Figure 1D). BCLA patients were then ranked according to the risk scores calculated based on the autophagy-related lncRNA prognosis signature (Figure 1E). The scatter dot plot showed that the survival rates of the BCLA patients correlated with the risk score according to the autophagy-related lncRNA prognostic signature; patients with a higher risk score demonstrated lower survival time (Figure 1F). The heatmap showed distinct differences in the levels of the 5 prognostic signature-related lncRNAs in the high- and low-risk BCLA patients. High-risk patients expressed higher levels of risk factors (AC108449.2 and LINC02178), while low-risk patients expressed higher levels of protective factors (Z83843.1, FAM13A−AS1 and USP30−AS1) (Figure 1G).

### Correlation analysis of the autophagy-related lncRNA prognosis signature with other clinicopathological parameters

**Figure 2:**
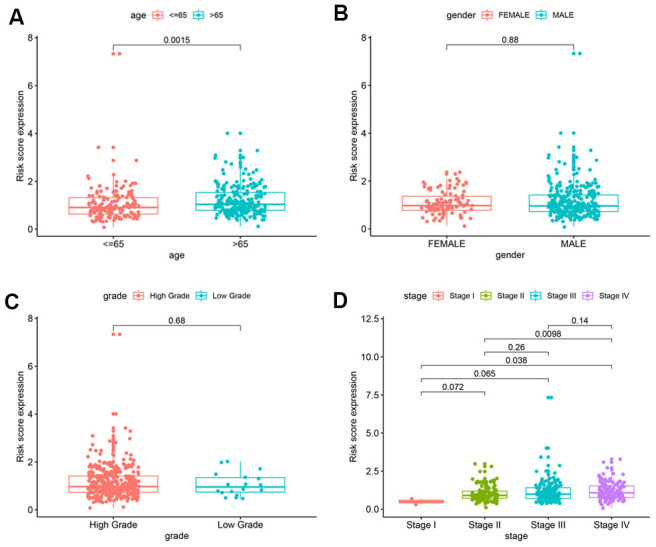
**Correlation analyses of the autophagy-related lncRNA prognostic signature with various clinicopathological characteristics of the BCLA patients.** The analysis compares the expression of the 5 prognostic lncRNAs in the BCLA patient cohort from the TCGA database stratified according to (**A**) age (< 65 y, n = 189; ≥ 65 y, n = 235); (**B**) gender (male, n = 291 vs. female, n = 102); (**C**) tumor grades (high grade, n = 372; low grade, n = 18); and (**D**) AJCC stages (stages I/II, n = 115; stages III/IV, n = 266).

We then analyzed the correlation between the risk scores from the autophagy-related lncRNA prognosis signature and the clinicopathological characteristics of the BCLA patients from TCGA database. Patients aged > 65 years showed significantly higher risk scores compared to patients aged ≤ 65 years (Figure 2A). The risk scores were statistically similar between the male and female BCLA patients (Figure 2B). Furthermore, the risk scores were statistically similar for BCLA patients belonging to high- and low-grades, probably because majority of the patients analyzed belonged to high-grade group (high-grade, n = 372; low-grade, n = 18; Figure 2C). Moreover, BCLA patients belonging to the higher AJCC stages showed higher risk scores than those with lower AJCC stages (Figure 2D). These results demonstrate that the autophagy-related lncRNA risk signature is associated with the clinicopathological characteristics of BCLA patients.

**Figure 3:**
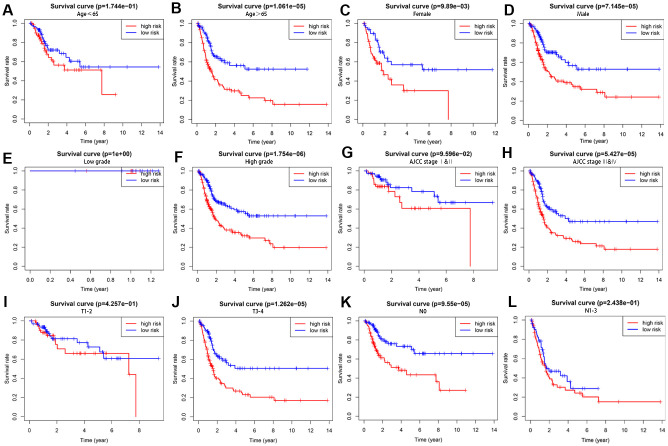
**The survival rates of high- and low-risk BCLA patients stratified by different clinicopathological characteristics.** Kaplan Meier survival curve analysis shows overall survival (OS) rates of high- and low-risk BCLA patients from the TCGA database stratified by (**A**, **B**) age (≤ 65 y vs. > 65 y), (**C**, **D**) gender (male vs. female), (**E**, **F**) tumor grades (high grade vs. low grade), (**G**, **H**) AJCC stages (stages I and II vs. stages III and IV), (**I**, **J**) T stages (T1/T2 vs. T3/T4), and (**K-L**) N stages (N0 vs. N1/N2/N3).

We further performed a stratification analysis to investigate the prognostic value of the autophagy-related lncRNAs. The patients were grouped according to age (≤ 65 and > 65), gender (female and male), tumor grade (low grade and high grade), AJCC stage (stages I and II and stages III and IV), T stage (T1/T2 and T3/T4) and N stage (N0 and N1/N2/N3). As shown in Figure 3, the Kaplan-Meier survival curve analysis showed that the OS rate was significantly shorter for the high-risk patients compared to the low-risk patients based on the prognostic signature among male patients (*P* = 7.145e−05), female patients (*P* = 9.89e−03), and those with age > 65 (*P* = 1.061e−05), high grade (*P* = 1.754e−06), AJCC stages III and IV (*P* = 5.427e−05),T3-4 stages (*P* = 1.262e−05) and N0 stage (*P* = 9.55e−05). However, the OS rate between the high- and low-risk groups based on the prognostic signature were similar for patients with ages ≤ 65 (*P* = 1.744e−01), low grade (*P* = 1e+00), AJCC stages I and II (*P* = 9.596e−02), T1-2 stages (*P* = 4.257e−01) and N1-3 stages (*P* = 2.438e−01), probably because of the smaller sample size. These results suggest that the prognosis signature can accurately determine the prognosis of patients relative to other clinicopathological characteristics.

### The autophagy-related lncRNA signature is an independent prognostic factor

**Figure 4:**
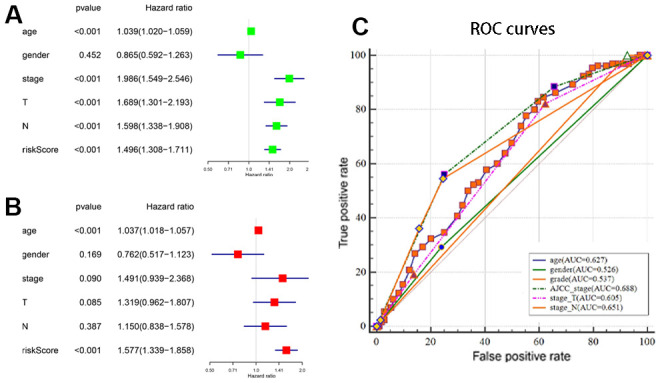
**Estimation of the prognostic accuracy of the autophagy-related lncRNA prognostic signature and other clinicopathological variables in the BCLA patients.** (**A**) Univariate Cox regression analysis shows the correlation between overall survival and various clinicopathological parameters such as age, gender, AJCC stage, T stage, N stage and the autophagy-related lncRNA prognostic signature risk score. The remaining parameters (*P* < 0.001) are significantly associated with OS in addition to the gender. (**B**) Multivariate Cox regression analysis shows that age and risk score (*P* < 0.001) are independent prognostic indicators for overall survival rates of BCLA patients. (**C**) Receiver operating characteristic (ROC) curve analysis shows the prognostic accuracy of clinicopathological parameters such as age, AJCC stage, T stage, N stage and autophagy-related lncRNA prognostic risk score.

Next, we performed univariate and multivariate Cox regression analyses to determine if the autophagy-related lncRNA prognostic signature was an independent prognostic factor for patients with BLCA. Univariate analyses showed that age (*P* < 0.001), AJCC stage (*P* < 0.001), T stage (*P* < 0.001), N stage (*P* < 0.001) and autophagy-related lncRNA prognostic risk score (*P* < 0.001) were significantly associated with OS (Figure 4A). The HR value tended to infinity within the tumor grade because of uneven distribution of samples (18 cases in low grade and 372 cases in high grade). Multivariate analyses showed that age (*P* < 0.001) and autophagy-related lncRNA prognostic risk score (*P* < 0.001) were significantly associated with OS (Figure 4B). As shown in Figure 4C, the ROC curve analysis demonstrated that the AUC value for the autophagy-related lncRNAs prognostic signature was 0.710, which was higher than the AUC values for age (AUC = 0.627), gender (AUC= 0.526), grade (AUC= 0.537), AJCC stage (AUC=0.688), T stage (AUC=0.605) and N stage (AUC= 0.651). These data demonstrate that the autophagy-related lncRNA prognostic signature is an independent prognostic factor for BLCA patients.

### Evaluation of the prognostic prediction nomogram that includes autophagy-related lncRNA prognostic signature risk score

**Figure 5:**
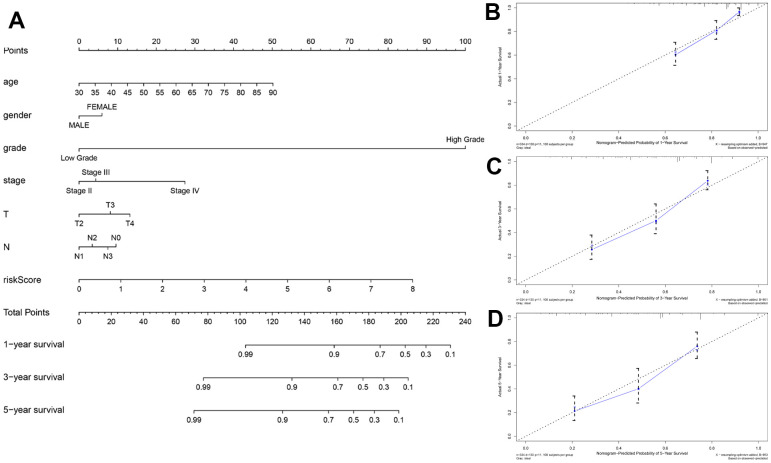
**Construction and validation of the prognostic nomogram with autophagy-related lncRNA prognostic signature risk score as one of the parameters.** (**A**) The predicted 1-, 3-, 5-year survival rates of BCLA patients based on the prognostic nomogram constructed using the risk score from autophagy-related lncRNA prognostic signature and clinicopathological parameters such as age, AJCC stage, T stage, N stage is shown. (**B**–**D**) Calibration curves show the concordance between predicted and observed (B) 1-year, (**C**) 3-year, and (**D**) 5-year survival rates of high- and low-risk BCLA patients based on the prognostic nomogram after bias correction.

Nomograms are commonly used tools used by clinicians to accurately predict survival time for a patient by calculating the nomogram score based on the points assigned for each prognostic factor included in the nomogram [23]. We constructed a nomogram to accurately estimate the 1-, 3-, and 5-year survival probabilities by using risk score calculated from the autophagy-related lncRNA prognostic signature and other clinicopathological factors, including age, gender, grade, AJCC stage, T stage and N stage (Figure 5A). The concordance index (C-index) value for the nomogram was 0.715. The calibration curve analysis showed that the actual and the predicted 1-, 3-, and 5-year survival times were in agreement when compared with the reference line (Figure 5B–5D). These results demonstrated that the nomogram using the autophagy-related lncRNA prognostic signature risk scores was reliable and accurate.

### Construction of the lncRNA–mRNA co-expression network and functional enrichment analysis

**Figure 6:**
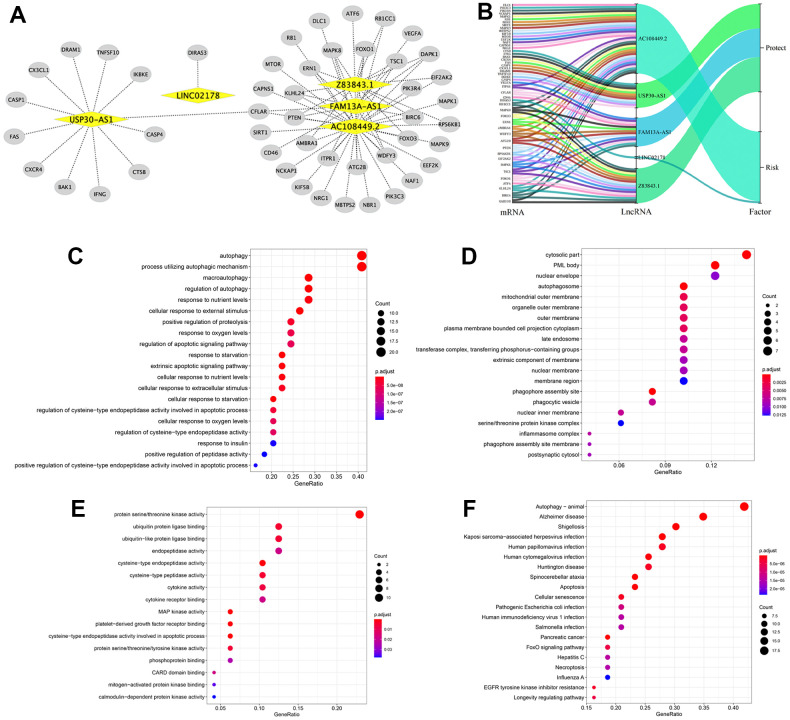
**Construction of the autophagy-related lncRNA–mRNA co-expression network and functional enrichment analyses.** (**A**) Diagrammatic representation of the autophagy-related lncRNA–mRNA network shows 77 lncRNA-mRNA co-expression pairs formed between 5 autophagy-related lncRNAs and 49 mRNAs. The yellow circles correspond to autophagy-related lncRNAs, and the gray diamonds correspond to the mRNAs. Every edge represents a co-expression relationship between an lncRNA and an mRNA in the context of BCLA. (**B**) The Sankey diagram shows the connection degree between the 49 mRNAs and 5 autophagy-related lncRNAs (risk/protective). (**C**–**E**) Gene Ontology (GO) analysis results show the enriched (**C**) biological processes, (**D**) cell components and (**E**) molecular functions associated with the mRNAs that co-express with the 5 autophagy-related lncRNAs. (**F**) Kyoto Encyclopedia of Genes and Genomes (KEGG) pathway analysis results shows the enriched signaling pathways associated with the mRNAs that co-express with the 5 autophagy-related lncRNAs.

Next, we investigated the potential functions of the 5 autophagy-related lncRNAs in BLCA by constructing the lncRNA-mRNA co-expression network using Cytoscape. The lncRNA-mRNA co-expression network contained 77 lncRNA-mRNA pairs based on the threshold parameters, Pearson correlation coefficient |R| > 0.3 and P < 0.05 (Figure 6A). Among these, 49 mRNAs significantly correlated with the 5 lncRNAs in the prognostic signature. The Sankey diagram showed the relationship between the 49 mRNAs and 5 lncRNAs (risk/protective) (Figure 6B). The top three GO terms for the biological processes were autophagy, process utilizing autophagic mechanism, and macroautophagy (Figure 6C). The top three GO terms for the cellular components were cytosolic part, PML body, and the nuclear envelope (Figure 6D). The top three GO terms for molecular functions were protein serine/threonine kinase activity, ubiquitin protein ligase binding, and ubiquitin−like protein ligase binding (Figure 6E). KEGG pathway analysis confirmed that autophagy was the most significantly enriched pathway (Figure 6F).

### Gene set enrichment analysis

**Figure 7:**
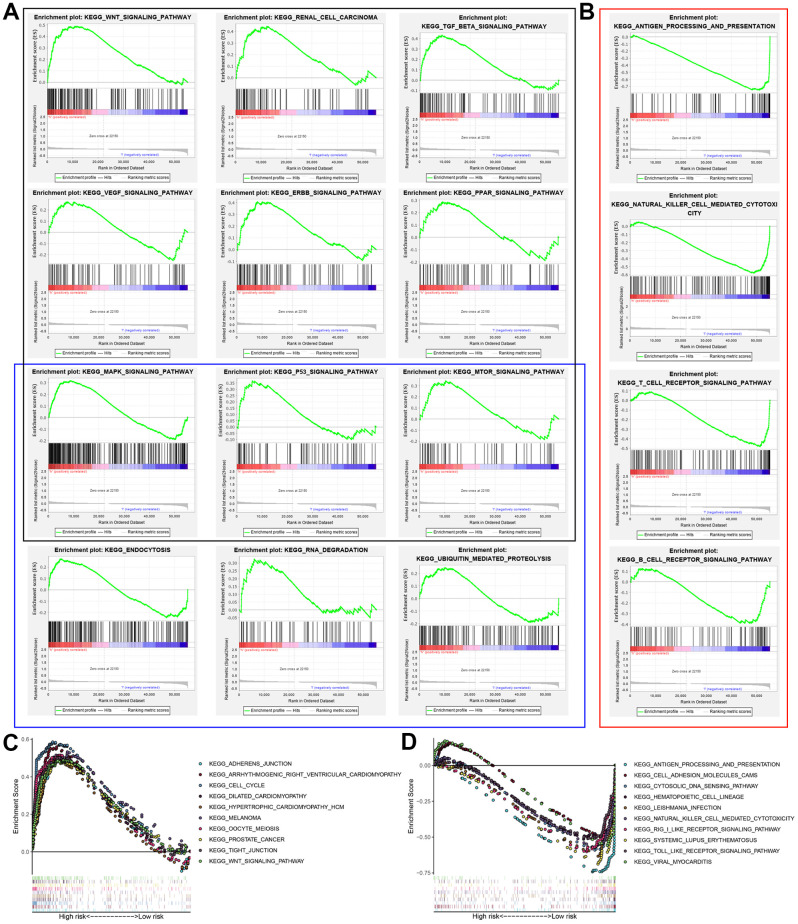
**Gene set enrichment analysis (GSEA) of high-risk and low-risk BCLA patients based on the autophagy-related lncRNA prognostic signature.** (**A**) GSEA results show significant enrichment of cancer- and autophagy-related signaling pathways in the high-risk BCLA patients. The black and blue boxes correspond to cancer-related and autophagy-related KEGG signaling pathways, respectively. (**B**) GSEA results show significant enrichment of immunoregulatory signaling pathways in the low-risk BCLA patients. (**C**, **D**) The top 10 KEGG signaling pathways in the (**C**) high-risk and (**D**) low-risk BCLA patients.

GSEA results showed that the altered genes in the high-risk BCLA patients belonged to pathways related to autophagy and cancer, WNT signaling pathway, renal cell carcinoma, TGF-βsignaling pathway, VEGF signaling pathway, ERBB signaling pathway, PPAR signaling pathway, MAPK signaling pathway, P53 signaling pathway, mTOR signaling pathway, endocytosis, RNA degradation and ubiquitin-mediated proteolysis (Figure 7A). Immunoregulatory pathways against cancer were significantly enriched in the low-risk group, including pathways related to antigen processing and presentation, natural killer (NK) cell-mediated cytotoxicity, T cell receptor (TCR) signaling, chemokine signaling and B cell receptor (BCR) signaling (Figure 7B). This suggested that activation of pathways regulation immune function in the low-risk group may contribute to positive prognosis or longer survival outcomes. The top 10 KEGG pathways in the high-risk and low-risk groups based on GSEA are shown in Figure 7C and 7D. These results suggested that a high prognostic signature risk score correlates with autophagy and cancer, whereas low prognostic signature risk score correlates with enhanced immune function. These data provided valuable insights for future investigations into potential individualized treatments for BLCA patients belonging to different risk groups.

## DISCUSSION

The most common malignancy of the urinary system is BLCA, whose incidence rates are constantly increasing worldwide [1, 3]. The prognosis of BCLA patients is poor because of late diagnosis and high rate of therapeutic resistance [24]. The role of autophagy in tumorigenesis has been reported for several cancers, including BLCA [25]. Therefore, autophagy-related biomarkers are potential diagnostic biomarkers and therapeutic targets for BCLA patients. Previous studies have focused on the role of specific autophagy-related genes in BCLA progression [26].

LncRNAs are a new class of non-coding RNA molecules that regulate cancer cell growth, progression, and survival [27]. Hence, they are potential biomarkers that can predict cancer risk and survival outcomes. In this study, we systematically analyzed the prognostic prediction accuracy of autophagy-related lncRNAs in BCLA using bioinformatics and statistical tools.

We first identified 7 autophagy-related lncRNAs that significantly correlated with OS based on the univariate Cox regression analysis of the expression of autophagy-related lncRNAs in the BCLA patient samples from the TCGA database. Furthermore, 5 autophagy-related lncRNAs, Z83843.1, LINC02178, FAM13A−AS1, USP30−AS1 and AC108449.2 were selected to construct a prognostic signature based on their performance in the multivariate Cox regression analysis. The risk score of each BCLA patient was calculated according to the expression of the five autophagy-related lncRNAs in the prognostic signature and patients were classified into high- and low- risk groups based on the median risk score. BCLA patients with high-risk scores showed shorter survival times compared to those with low-risk scores. Principal components analysis (PCA) based on the confirmed five autophagy-related lncRNAs showed two significantly different distribution patterns between high-risk and low-risk groups. ROC curve analysis validated the prognostic accuracy of the autophagy-related lncRNA prognostic signature in the BLCA patients. The risk score based on the autophagy-related lncRNA prognostic signature was an independent prognostic factor based on multivariate Cox regression analysis. Stratified correlation analysis showed that the autophagy-related lncRNA prognostic signature accurately predicted survival outcomes for the high- and low-risk BCLA patients.

The autophagy-related lncRNA prognostic signature performed more reliably than the other traditional clinical indicators in prognostic prediction. A nomogram is an effective and reliable clinical tool to predict survival of cancer patients [28]. Therefore, we developed a robust nomogram consisting of several clinical variables (age, gender, grade, AJCC stage, T stage and N stage) and the risk scores based on the autophagy-related lncRNA prognostic signature to improve prognostic prediction of BCLA patients. Older patients (age ≥ 60 years) and those with higher tumor grades and advanced stages are usually associated with worse cancer prognosis, which is consistent with our results. Moreover, calibration plots demonstrated that the actual and predicted 1-, 3-, and 5-year survival rates based on the nomogram were similar. Overall, the autophagy-related lncRNA prognostic signature accurately predicts survival outcomes of BLCA patients in our study and shows great potential for clinical applications, including individualized prognosis and therapy.

Autophagy is a highly conserved intracellular catabolic process involved in the phagocytosis and degradation of abnormal organelles, proteins and pathogens through the lysosomal pathway [29]. The role of autophagy in cancer is controversial because it can play both tumor suppressor and oncogenic functions [30]. During early stages of tumor development, autophagy-related cell death can suppress tumor progression; autophagic dysregulation can also induce genomic instability and necrosis-induced inflammation, both of which promote tumor growth [31]. Conversely, autophagy sustains tumor metabolism, growth, and survival in nutrient-deprived conditions in the tumor microenvironment and contributes to drug resistance during tumor metastasis [32]. Autophagy is regulated by several signaling pathways, including the PI3K/AKT/mTOR signaling pathway [33] and ubiquitin-proteasome system (UPS). [34] In recent years, several lncRNAs have been implicated in the regulation of cell growth and survival by directly targeting autophagy-related genes. For example, Wang et al. reported that lncRNA ATB induced autophagy by enhancing the expression of autophagy-related protein 5 (ATG5) through activation of the Yes-associated protein (YAP) in hepatocellular carcinoma (HCC) cells [35]. We identified the genes whose expression is regulated by each of the 5 autophagy-associated lncRNAs in BLCA and constructed the lncRNA-mRNA co-expression network. GO, KEGG, and GSEA functional enrichment analyses showed that autophagy-related GO terms or signaling pathways were enriched. GSEA analyses also revealed distinct differences in the autophagy-related signaling pathways between the high- and low-risk groups. Several cancer- and autophagy-related pathways were enriched in the high-risk group, whereas immunomodulatory pathways were enriched in the low-risk group. This suggested that increased immunity correlates with improved prognosis. These results were concordant with the current understanding that autophagy is a critical modulator of BLCA progression [12, 13].

There are several limitations in our study. Firstly, our findings need to be further validated in other independent cohorts to determine the robustness of the autophagy-related lncRNA prognostic signature. Secondly, our study was based on a single cohort of 409 patients from the publicly available TCGA database. Moreover, samples belonging to BCLA patients with high-grade tumor (n = 385) were significantly larger than those with low-grade tumors (n = 21), which may have skewed our results and hence need to be further analyzed with larger and more even number of samples in the high-risk and low-risk groups. Finally, further investigations involving biochemical experiments such as immunohistochemistry, quantitative real-time PCR, and flow cytometry, and clinical data analyses are required to further confirm our findings.

In conclusion, our study showed that the autophagy-related lncRNA prognostic signature accurately predicts the survival outcomes of BCLA patients with BLCA and distinguishes them into high- and low-risk groups. We also established and validated a prognostic nomogram by combining the autophagy-related lncRNA prognostic signature and other clinicopathological features. Our study demonstrated that this nomogram can provide an individualized and accurate survival prediction. Our study also suggests that these 5 autophagy-related lncRNAs are potential prognostic and diagnostic biomarkers as well as promising targets for BCLA therapy.

## MATERIALS AND METHODS

### Patient data acquisition

The raw RNA-sequencing (RNA-seq) data and clinical information of 409 BLCA patients was downloaded from The Cancer Genome Atlas (TCGA) data portal (https://tcgadata.nci.nih.gov/tcga/). The Ensembl human genome browser, GRCh38.p13 (http://asia.ensembl.org/index.html) was used to annotate and classify the lncRNAs and protein-coding genes [36]. Patient samples were excluded (n = 16) if survival times of patients were less than 30 days to eliminate non-cancer related deaths. In addition, patients with incomplete clinical data (grade stage, n = 3; AJCC stage, n = 2) were excluded from the study. Since the data was obtained from a public database, approval from the Ethics committee or written informed consent from patients was not required.

### Identification of autophagy-related lncRNAs

We first identified 232 autophagy-associated genes from the Human Autophagy Database (HADb; http://www.autophagylu/index.html), which contains exhaustive, up-to-date list of human autophagy-related genes [37]. We calculated Pearson correlation coefficients to determine the correlation between the expression of the lncRNAs and the corresponding autophagy-related genes. The autophagy-related lncRNAs were selected based on the criteria that the absolute value of correlation coefficient was greater than 0.7 (|R|>0.7) and the *P* value was less than 0.05 (P < 0.05).

### Construction of the prognostic signature

The univariate Cox regression model was used to identify autophagy-related lncRNAs whose expression levels were significantly associated (*P* < 0.05) with the overall survival (OS) of the BLCA patient cohort. The hazard ratios (HRs) were used to identify risk-related lncRNAs (HR > 1) and protective lncRNAs (HR < 1). Subsequently, the candidate autophagy-related lncRNAs were subjected to multivariate Cox regression analysis to evaluate their contribution as independent prognostic factors in patient survival. Thus, we identified five target autophagy-related lncRNAs as candidates for the prognostic signature model, which was constructed based on a linear combination of the lncRNA expression levels and regression coefficients obtained from the multivariate Cox regression model. The optimal lncRNA prognostic signature was selected based on the lowest Akaike information criterion (AIC) value for further analysis. The computational formula used to determine the risk score for each patient based on this prognostic signature model was as follows: $\text{Risk Score}=\sum_{i=1}^{n}Coef\left(i\right)\times x(i)$, where *Coef* (*i*) and *x*(*i*) represent the estimated regression coefficient and the expression value of each autophagy-related lncRNA, respectively.

### Evaluation of the prognostic signature

The BCLA patients were classified into high-risk or low-risk groups based on their prognostic risk score by using the median risk score as a cut-off point. The Kaplan–Meier survival curve and two-sided log-rank test was used to compare the overall survival (OS) of the high- and low-risk group patients. Principal component analysis (PCA) was performed to visualize gene expression patterns in the patient samples from the two groups. The receiver-operating characteristic (ROC) curves were applied to evaluate the diagnostic efficacy of each clinicopathological characteristic and the prognostic signature. Stratified survival analysis was performed to examine the accuracy of the prognostic signature in predicting patient survival outcomes. Furthermore, univariate and multivariate Cox regression analyses were performed to evaluate whether the risk score was independent of other clinical variables such as age, gender, grade, AJCC stage, T stage and N stage in determining the prognosis of the BCLA patients. M stage was not analyzed because the data was missing for several patients. *P* < 0.05 was considered statistically significant.

### Establishment and validation of nomogram

We constructed a nomogram by integrating traditional clinical variables such as age, gender, grade, AJCC stage, T stage and N stage as well as the risk score derived from the prognostic signature to analyze the probable 1-, 3-, and 5-year OS of the BLCA patients. We then used the concordance index (C-index) to evaluate the discrimi-nation and predictive ability of the nomogram. The range of the C-index value was 0.5 to 1.0. A higher C-index indicates greater discrimination ability of the predicting model. Furthermore, calibration curves of the nomogram were generated to examine the concordance between pre-dicted survival and observed survival after bias correction.

### Construction of the LncRNA-mRNA co-expression network

The mRNA-lncRNA co-expression network was constructed to analyze the correlation between the autophagy-related lncRNAs and their target mRNAs. Pearson correlation coefficients were calculated to identify the mRNAs that are significantly associated with their target lncRNAs based on the absolute threshold coefficient value > 0.3. The lncRNA-mRNA co-expression network was constructed and visualized using the Cytoscape software (version 3.7.2, http://www.cytoscape.org/).

### Functional enrichment analysis

The lncRNA-related mRNAs were subjected to gene ontology (GO) enrichment analysis to identify the biological processes, molecular functions, and cellular components associated with the lncRNAs. The Kyoto Encyclopedia of Genes and Genomes (KEGG) pathway analysis was used to determine the main signaling pathways regulated by these lncRNAs. P < 0.05 was considered statistically significant.

### Gene set enrichment analysis (GSEA)

The genome wide expression profiles of the BCLA patients were subjected to gene set enrichment analysis (GSEA; http://www.broadinstitute.org/gsea) to determine the genes that are differentially expressed between the high- and low-risk group patients [38]. The gene sets were filtered using the maximum and minimum gene set size of 500 and 15 genes, respectively. The enriched gene sets were obtained based on a *P* value < 0.05 and a false discovery rate (FDR) value < 0.25 after performing 1,000 permutations.

### Statistical analysis

The data was processed using the PERL programming language (Version 5.30.2, http://www.perl.org). All statistical analyses were performed using the R software (version 3.6.2, https://www.r-project.org/). P < 0.05 was regarded as statistically significant.
